# SpineMAE: A bone‐window self‐supervised and structure‐aware framework for 3D cervical vertebra segmentation and fracture classification

**DOI:** 10.1002/acm2.70575

**Published:** 2026-05-04

**Authors:** Qing Liang, Jingding Zhao, Fang Yang, Lin Shi, Yang Song, Xianjun Chen, Zewen Shi, Qingjiang Pang

**Affiliations:** ^1^ Hangzhou Medical College Hangzhou Zhejiang Province China; ^2^ Health Science Center Ningbo University Ningbo China; ^3^ Ningbo No. 2 Hospital Wenzhou Medical University Ningbo China; ^4^ College of Science and Technology Ningbo University Ningbo China

**Keywords:** cervical spine fracture, fracture classification, medical image analysis, self‐supervised learning, vertebral segmentation

## Abstract

**Introduction:**

Accurate segmentation and classification of cervical spine fractures are essential for timely diagnosis and clinical decision‐making in trauma care. Existing deep learning approaches often require extensive manual annotations and struggle to maintain anatomical consistency across vertebral levels, limiting their reliability and generalization.

**Methods:**

We propose a unified three‐stage framework that integrates self‐supervised representation learning, structure‐aware segmentation, and vertebra‐level classification. A bone‐window 3D masked autoencoder first pretrains the encoder to learn bone‐sensitive volumetric features from unlabeled CT scans. The pretrained encoder is then transferred to a structure‐aware 3D U‐Net that incorporates bone‐likelihood and edge priors with geometry‐consistency regularization for anatomically coherent vertebral segmentation. Finally, a vertebra‐level classification head performs fracture prediction using ROI features with positional embeddings to encode anatomical context.

**Results:**

Evaluated on the RSNA Cervical Spine Fracture Detection dataset, the proposed method achieved a Dice score of 89.23% for cervical vertebra segmentation. For vertebra‐level fracture classification, it achieved an AUC of 0.969 with sensitivity of 0.788 and specificity of 0.962 (macro‐averaged across C1–C7), outperforming CNN‐ and transformer‐based baselines.

**Conclusions:**

The proposed framework provides a robust, interpretable, and data‐efficient solution for automated cervical spine fracture detection and localization. By combining self‐supervised 3D pretraining with anatomy‐aware modeling, it offers a practical pathway toward clinically reliable AI‐assisted diagnosis in spinal imaging.

## 1 INTRODUCTION

Cervical spine fractures are among the most critical injuries in trauma imaging, often caused by high‐energy impacts such as traffic accidents or falls, and occasionally by low‐energy mechanisms in elderly patients with osteoporosis. These injuries pose a serious risk of neurological damage and permanent disability, making rapid and accurate detection essential for clinical management.^1^, ^2^, ^3^ Computed tomography (CT) remains the gold standard for diagnosis due to its high spatial resolution and bone sensitivity. However, manual inspection of 3D CT volumes is labor‐intensive and prone to variability, especially when fractures are subtle or occur near complex anatomical regions such as C1–C2 or the cervicothoracic junction. This motivates the development of automated, reliable, and interpretable algorithms to assist radiologists in identifying and localizing fractures efficiently. The RSNA Cervical Spine Fracture Detection benchmark has stimulated rapid progress in automated fracture detection, but existing high‐performing solutions are often evaluated at the patient level and may provide limited localization interpretability.

Deep learning has revolutionized medical image analysis, achieving remarkable progress in segmentation and classification. Convolutional neural networks (CNNs), particularly 3D U‐Net variants, have become the backbone of most medical segmentation systems but still struggle to capture long‐range anatomical context, leading to fragmented or inconsistent vertebral predictions. Transformer‐based architectures such as UNETR and Swin UNETR improve contextual modeling through global attention, yet their reliance on large‐scale voxel‐level annotations limits scalability and cross‐domain robustness.^4^, ^5^, ^6^

Moreover, most existing approaches treat vertebral segmentation and fracture classification as separate tasks, missing the synergy between structural localization and semantic diagnosis. Few studies explore self‐supervised pretraining or anatomical priors to reduce annotation dependency and enhance interpretability.^7^, ^8^, ^9^ Recently, masked autoencoders (MAE) have shown strong potential for learning domain‐specific representations without labels, which can capture cortical and trabecular bone features critical for subtle fracture detection.^10^, ^11^, ^12^, ^13^ In addition, several competition‐driven fracture detectors focus on classification without producing vertebra masks, which limits visual verification and error analysis in clinical practice.

To address these challenges, we propose SpineMAE, a unified three‐stage framework for precise cervical spine fracture segmentation and classification. First, a bone‐window self‐supervised MAE pretrains the encoder on unlabeled 3D CT scans to learn bone‐sensitive representations. Second, a structure‐aware 3D U‐Net leverages bone‐likelihood and edge priors with gated attention and geometry‐consistency regularization for anatomically coherent vertebral segmentation. Finally, a vertebra‐level classifier uses ROI features and positional embeddings to encode anatomical hierarchy for consistent fracture prediction.^14^, ^15^, ^16^

Extensive experiments on the RSNA Cervical Spine Fracture Detection dataset demonstrate that SpineMAE outperforms both CNN‐ and transformer‐based baselines, achieving superior segmentation and classification performance. In summary, our contributions are threefold:

1. an innovative bone‐window self‐supervised pretraining strategy for learning bone‐sensitive 3D representations;
2. a structure‐aware segmentation network with geometry‐consistency regularization; and
3. comprehensive experimental validation showing consistent superiority over state‐of‐the‐art baselines.

Together, these advances establish a robust, data‐efficient, and interpretable pipeline for cervical spine fracture analysis, offering a practical step toward clinical deployment in computer‐assisted radiology.^17^, ^18^, ^19^, ^20^

## 2 METHODS

**FIGURE 1:**
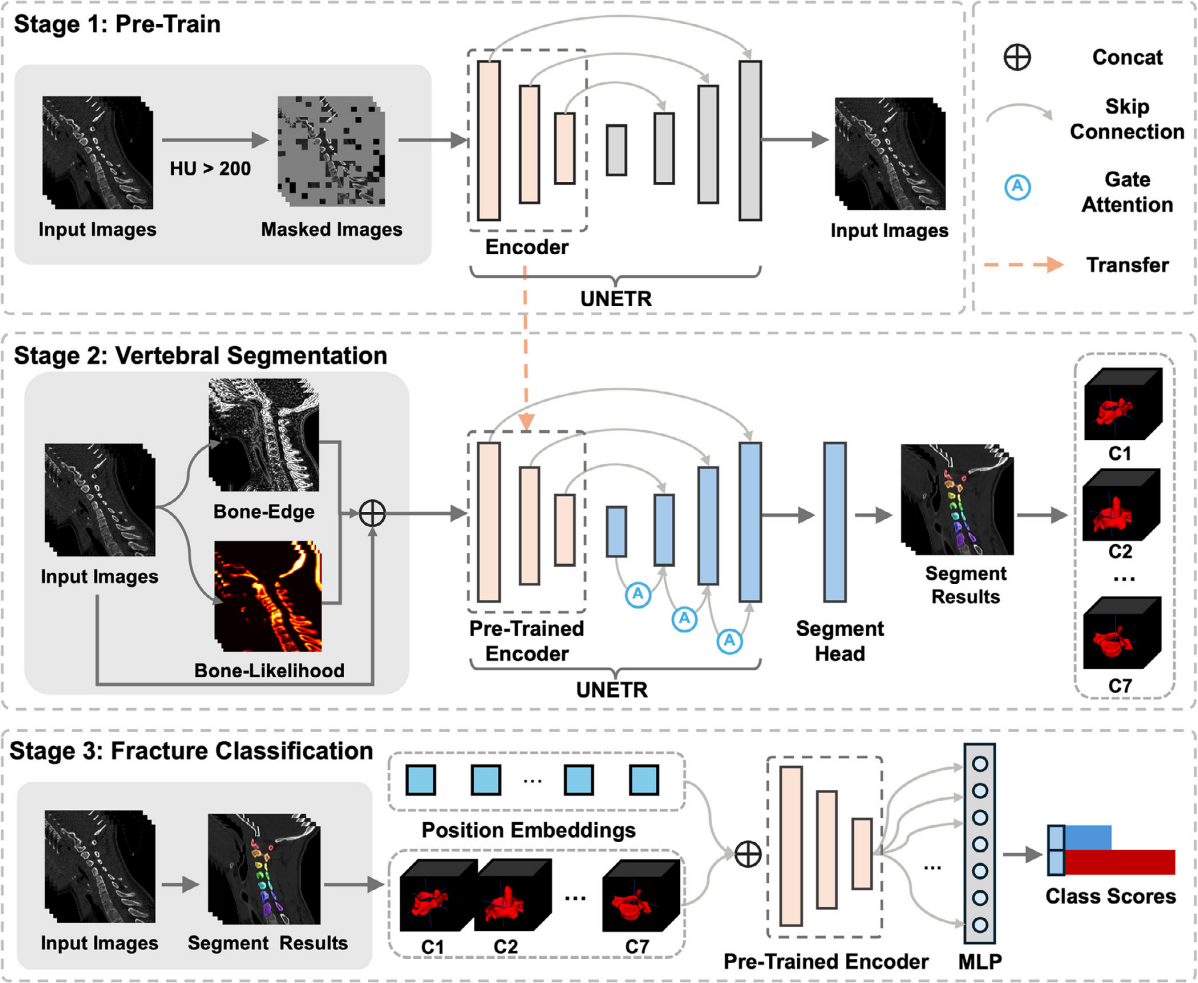
Overview of the proposed three‐stage framework SpineMAE. The model integrates self‐supervised pretraining, vertebral segmentation with structural priors, and vertebra‐level fracture classification.

Our framework (Figure 1) is a three‐stage pipeline that couples bone‐sensitive self‐supervised pretraining with structure‐aware 3D segmentation and vertebra‐level fracture classification. Given a preprocessed cervical CT volume $V\in R^{H\times W\times D}$, Stage 1 learns a generic encoder tuned to cortical bone morphology; Stage 2 predicts multi‐class masks for background and C1–C7 while enforcing geometric plausibility; Stage 3 crops standardized vertebra ROIs and predicts vertebra‐wise fracture probabilities. All stages are implemented in PyTorch and share the same 3D encoder initialized from Stage 1.

### 2.1 Bone‐window self‐supervised pretraining

Stage 1 follows a masked‐autoencoding paradigm tailored to bone‐windowed CT volumes, with an intensity‐aware masking scheme that reduces the chance of hiding cortical regions. Each volume is windowed with a bone window of *WL* = 300 *HU* and *WW* = 1500 *HU*, corresponding to clipping intensities to [−450,1050] *HU*, linearly rescaled to [0,1] as $x_{CT}=\left(\mathrm{clip}(I_{HU},-450,1050)+450\right)\;/1500$, resampled to a spacing of 1.0 × 1.0 × 2.0 mm, and partitioned into nonoverlapping cubic patches of size $16^{3}$ voxels for masked autoencoding pretraining. We form tokens by a linear projection and add a learnable 3D positional embedding. To bias learning toward cortical bone, masks are sampled with a target ratio of 70% using a patch‐level prior derived from the proportion of high‐HU voxels (threshold = 400 *HU*); bone‐rich patches therefore have a lower masking probability, so the encoder more frequently observes cortical structures while still reconstructing diverse anatomical contexts. A UNETR‐style ViT encoder processes only the visible tokens, and a lightweight decoder reconstructs the missing ones; positional embeddings are trilinearly resized when transferring across fields‐of‐view or slice thicknesses.

This design is motivated by the fact that vertebral boundaries and fracture‐related cues are predominantly expressed in the cortical shell and endplates, which contain high‐frequency texture information that is critical for downstream segmentation and subtle fracture assessment. Purely random masking may frequently occlude these thin cortical regions, encouraging the encoder to rely on lower‐frequency context. By reducing the masking probability of bone‐rich patches while maintaining a high overall masking ratio, the encoder learns more bone‐sensitive representations without sacrificing global anatomical context.

We keep Transformer math to a single, canonical multi‐head self‐attention expression. For input $Z\in R^{N_{v}\times d}$ at a given layer, the attention is

(1) $$\mathrm{MSA}\;\left(Z\right)=\;\mathrm{Concat}{\left(\mathrm{softmax}\left(\frac{Q_{h}K_{h}^{T}}{\sqrt{d_{h}}}\right)V_{h}\right)}_{h=1}^{H}W_{o},$$

where $Q_{h}=ZW_{h}^{Q}$, $K_{h}=ZW_{h}^{K}$, $V_{h}=ZW_{h}^{V}$, H is the number of heads, $d_{h}=d/H$, and $W_{o}$ is the output projection. The reconstruction objective is evaluated only on masked patches:

(2) $$L_{\mathrm{MAE}}=\frac{1}{\left|M\right|}\;\sum_{i\in M}{∥x_{i}-{\hat{x}}_{i}∥}_{2}^{2},$$

with M the masked index set and $x_{i}$, ${\hat{x}}_{i}$ the ground‐truth and reconstructed patch intensities. After convergence on unlabeled CTs, we transfer the encoder to downstream tasks. During downstream fine‐tuning (Stages 2 and 3), the lowest encoder blocks are frozen for several epochs to preserve low‐level bone cues, then progressively unfrozen for end‐to‐end optimization.

### 2.2 Structure‐aware vertebral segmentation

Stage 2 predicts background and C1–C7 masks from a three‐channel input that encodes explicit anatomical priors. The first channel is the same bone‐window CT image used in Stage‐1 pretraining. Specifically, each resampled scan is windowed with *WL* = 300 *HU* and *WW* = 1500 *HU*, corresponding to clipping intensities to [−450,1050] HU, and then linearly rescaled to [0,1] as $x_{CT}=\left(\mathrm{clip}(I_{HU},-450,1050)+450\right)\;/1500$. The second channel is a bone‐likelihood map obtained by linearly mapping HU values in [100,400] to [0,1], clipping values outside this interval, and applying light 3D Gaussian smoothing (σ=1 voxel) to suppress noise while preserving trabecular and cortical regions. The third channel is an edge‐enhancement map computed by applying a 3D Laplacian‐of‐Gaussian filter (σ=1 voxel) to the first channel, taking the absolute response, and min‐max normalizing it to [0, 1]. We stack the three volumes along the channel dimension to form $X_{seg}\in R^{3\times H\times W\times D}$.

The three channels are transformed differently because they encode different but complementary information. The first channel preserves the native bone‐window CT appearance and matches the input distribution used during Stage‐1 pretraining. The second and third channels are deterministic auxiliary priors derived from the same CT scan: the bone‐likelihood channel emphasizes bone density cues, whereas the edge‐enhancement channel emphasizes cortical boundaries and inter‐vertebral interfaces. Because these auxiliary priors have value distributions that differ from raw CT intensity, they are normalized separately to comparable numerical ranges before concatenation. During Stage‐2 fine‐tuning, we adopt a staged optimization strategy: the lower encoder blocks are initially frozen for several epochs and then progressively unfrozen, allowing the network to adapt to the auxiliary prior channels while preserving pretrained low‐level bone‐sensitive representations.

We adopt a UNETR‐style 3D U‐Net whose encoder is initialized from Stage 1. Multi‐scale encoder features are bridged to the decoder through skip connections but, before concatenation, they are filtered by gated attention modules that suppress irrelevant activations and sharpen boundaries along the upsampling path. Let $x^{l}$ be the skip feature at level l and $g^{l}$ the decoder gating signal at the same scale; attention coefficients are computed by additive gating:

(3) $$\begin{array}{cc} & q^{l}=\theta_{x}^{l}*x^{l}+\theta_{g}^{l}*g^{l}+b^{l},\\ & \alpha^{l}=\sigma \left(\psi^{l⊤}\text{ReLU}\left(q^{l}\right)\right),\;{\hat{x}}^{l}=\alpha^{l}⊙x^{l} \end{array}$$

where ∗ is a 1×1×1 convolution, σ the sigmoid, and ⊙ element‐wise multiplication. The filtered ${\hat{x}}^{l}$ is concatenated with the upsampled decoder feature and processed by a residual up‐block, improving separation between adjacent vertebrae and reducing spurious responses near osteophytes or ribs.

Given the limited number of pixel‐level labels, we combine supervised learning with morphology‐consistent self‐training. A seed model trained on the 87 labeled cases generates pseudo‐masks on non‐voxel‐labeled CT studies; only components exceeding vertebra‐specific minimum volumes and with high mean softmax probability are retained. Labeled and pseudo‐labeled cases are then mixed with confidence‐based sampling, and the model is retrained for one to two iterations. The segmentation objective integrates appearance, boundary, and geometric regularizers:

(4) $$\begin{array}{cc} L_{\mathrm{seg}} & =\sum_{c}w_{c}{\left(1-\mathrm{Dic}e_{c}\right)}^{\gamma }+\alpha L_{\mathrm{WCE}}+\beta L_{\mathrm{Boundary}}\\ & \;+\lambda_{\mathrm{ord}}\;L_{\mathrm{ord}}+\lambda_{\mathrm{ovl}}L_{\mathrm{ovl}}, \end{array}$$

where $L_{\mathrm{Boundary}}$ aligns predictions with the edge‐enhancement signal, $L_{\mathrm{ord}}$ softly enforces cranio‐caudal centroid ordering $z_{k}<z_{k+1}$, and $L_{\mathrm{ovl}}$ penalizes overlaps of adjacent labels. Post‐processing keeps the largest component per vertebra and computes centroid, principal axis, and an oriented bounding box, which define standardized ROIs resampled to a common physical size for Stage 3.

### 2.3 Vertebra‐level fracture classification

After Stage 2, each vertebra is represented by a standardized ROI cropped from the oriented bounding box and resampled to a fixed physical field‐of‐view and voxel spacing. Only the same bone‐windowed and linearly rescaled CT intensity used in Stages 1 and 2 is used for classification, and the encoder pretrained in Stage 1 is reused to extract a compact 3D feature $z_{k}$ for level k∈{1,…,7}. This reuse preserves bone‐sensitive cues while keeping the classifier lightweight and clinically practical.

(5) $$z_{k}^{\prime }=\left[z_{k}\;\left|\right|\;e_{k}\right],$$

To inject explicit anatomical identity, a learnable level embedding $e_{k}$ is assigned to each vertebral level (C1–C7) and concatenated with the ROI feature $z_{k}$. The operator [·||·] denotes vector concatenation. This design informs the classifier “which vertebra it is seeing,” leveraging the systematic morphological differences between levels (e.g., ringlike C1/C2 vs. more blocklike lower cervical vertebrae).

(6)

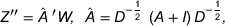




To promote inter‐level consistency, we optionally apply a single message‐passing step across the seven vertebral embeddings. Here $Z^{\prime }$ stacks the seven vectors 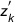 row‐wise, A is the chain adjacency (C1↔C2↔…↔C7), I is the identity (self‐loops), D is the degree matrix, and W is a learnable weight matrix. The normalized operator $\hat{A}$ diffuses cues along the cranio‐caudal axis, suppressing isolated false positives and stabilizing borderline cases that tend to co‐occur across adjacent levels.

(7)

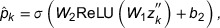




Each refined embedding 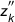 (the k‐th row of $Z^{\prime \prime }$) is fed to a two‐layer MLP to produce the fracture probability ${\hat{p}}_{k}$. The matrices $W_{1}$, $W_{2}$ and bias $b_{2}$ are learnable parameters, ReLU(·) is the activation, and σ(·) is the sigmoid. We apply dropout in the MLP for regularization and use balanced mini‐batches so that fractured and non‐fractured ROIs contribute comparably to the gradient.

(8) $$L_{\mathrm{cls}}=-\frac{1}{N}\sum_{k=1}^{7}\left(y_{k}\log{\hat{p}}_{k}+\left(1-y_{k}\right)\log\left(1-{\hat{p}}_{k}\right)\right),$$

Training uses a binary cross‐entropy loss over the seven vertebrae, where $y_{k}\in 0,1$ is the ground truth (GT) label and N is the number of vertebra instances in the mini‐batch.

## 3 RESULTS

### 3.1 Datasets

We evaluate on the RSNA Cervical Spine Fracture Detection dataset, which comprises multicenter cervical CT studies stored as DICOM series and accompanied by structured labels. The training set contains 2,019 studies. Pixel‐wise annotations are available for 87 cases as NIfTI masks. All segmentation training/evaluation and vertebra‐level fracture classification in this study are conducted on these 87 voxel‐labeled studies (i.e., 609 vertebra ROIs), while the remaining scans are used only for Stage‐1 self‐supervised pretraining. The file train provides a patient‐level label and vertebra‐level binary labels for C1–C7; train_bounding_boxes supplies slice‐level bounding boxes (7218 rows) that localize suspicious regions; test and sample_submission define the submission schema. At the patient level, the label distribution is moderately imbalanced (no‐fracture 52.4% and fracture 47.6%). At the vertebra level, fractures are relatively rare (85/609, 14.0%), resulting in a substantially more imbalanced classification problem. Table 1 summarizes the vertebra‐level label distribution for one representative fold of the patient‐wise fivefold cross‐validation, illustrating the approximate train/test class distribution under the evaluation protocol. We use balanced mini‐batches during training to mitigate this imbalance. Labels exhibit frequent multi‐label co‐occurrence across vertebrae, and C7‐only fractures are the single most common pattern (223 cases). Heterogeneity in slice thickness and vendor protocols makes the benchmark suitable for assessing cross‐domain robustness in 3D medical imaging.

**TABLE 1:** Vertebra‐level label distribution (fractured vs. non‐fractured) for C1–C7 in one representative fold of the patient‐wise fivefold cross‐validation (counts shown as *F*/*NF*).

Vertebra	Train (*F*/*NF*)	Test (*F*/*NF*)	Total (*F*/*NF*)	Fracture % (total)
C1	4/66	1/16	5/82	5.7%
C2	10/60	3/14	13/74	14.9%
C3	9/61	2/15	11/76	12.6%
C4	8/62	1/16	9/78	10.3%
C5	12/58	2/15	14/73	16.1%
C6	17/53	3/14	20/67	23.0%
C7	10/60	3/14	13/74	14.9%
Total	70/420	15/104	85/524	14.0%

#### 3.1.1 Data preprocessing

All DICOM series are sorted by ImagePositionPatient/InstanceNumber and reconstructed into 3D volumes, then resampled to 1.0 × 1.0 × 2.0 mm spacing. For the raw CT channel used in Stages 1 and 2, intensities are windowed with a bone window of *WL* = 300 *HU* and *WW* = 1500 *HU*, clipped to [−450,1050] HU, and linearly rescaled to [0,1] using $x_{CT}=\left(\mathrm{clip}(I_{HU},-450,1050)+450\right)/1500$. No *z*‐score normalization is used for this channel. Two auxiliary channels are then computed from the same CT scan to inject anatomical priors used by the segmentation network: a bone‐likelihood map obtained by linearly mapping *HU* values in [100,400]−[0,1], clipping values outside this interval, and applying light 3D Gaussian smoothing (σ = 1 voxel); and an edge‐enhancement map obtained by applying a 3D Laplacian‐of‐Gaussian filter (σ = 1 voxel) to the raw CT channel, taking the absolute response, and min‐max normalizing it to [0,1]. Non‐body air is removed by thresholding and largest‐component selection to reduce computation and stabilize the auxiliary prior maps, but no body‐mask statistics are used in the normalization of the raw CT channel. Stage‐1 pretraining samples random 96×96×96 patches with a bias toward bone‐rich regions; Stage‐2 segmentation consumes the three‐channel volumes; and Stage‐3 classification uses Stage‐2 geometric anchors (centroid, principal axis, and oriented bounding box) to crop per‐vertebra ROIs normalized to a fixed physical field of view and spacing. Online 3D augmentations (small rotations, flips, mild elastic deformations, and contrast jitter) are applied during training.

#### 3.1.2 Data splitting

All splits were patient‐wise to prevent information leakage across slices or series from the same subject. For the 87 voxel‐wise annotated studies, we used stratified patient‐wise fivefold cross‐validation to preserve per‐vertebra (C1–C7) prevalence across folds, while lightly balancing slice thickness and scanner metadata to mitigate domain shift. In each fold, approximately 80% of studies were used for training/validation and 20% for testing. Pseudo‐masks for semi‐supervised segmentation were generated only on the training portion of each fold and were never used for validation or testing. Stage‐1 self‐supervised pretraining was performed on all available training CT studies (including those without voxel‐wise masks). For robustness, vertebral segmentation performance was reported as the mean ± standard deviation across the five folds, whereas vertebra‐level fracture classification results (including ROC and PR curves, as well as the confusion matrix) were obtained by aggregating the unseen test predictions from all five folds, yielding cross‐validated predictions for all 609 vertebra ROIs.

### 3.2 Evaluation metrics

We report segmentation and vertebra‐level classification metrics. Unless otherwise noted, segmentation counts (TP, FP, and FN) are computed at the voxel level per class and macro‐averaged across C1–C7; classification counts are computed per vertebra ROI. Thresholds for classification are calibrated on the validation set.

1. Accuracy

Measures the proportion of correct predictions:

(9) $$\mathrm{Accuracy}=\frac{\mathrm{TP}+\mathrm{TN}}{\mathrm{TP}+\mathrm{TN}+\mathrm{FP}+\mathrm{FN}},$$

2. Precision

Reliability of positive predictions:

(10) $$\mathrm{Precision}=\frac{\mathrm{TP}}{\mathrm{TP}+\mathrm{FP}},$$

3. Recall

Ability to detect positives:

(11) $$\mathrm{Recall}=\frac{\mathrm{TP}}{\mathrm{TP}+\mathrm{FN}}\;,$$

4. F1‐score

Harmonic mean of Precision and Recall:

(12) $$\text{F1}=\frac{2\cdot \text{Precision}\cdot \text{Recall}}{\text{Precision}+\text{Recall}},$$

5. Dice coefficient

Spatial overlap between prediction and GT:

(13) $$\text{Dice}=\frac{2\cdot \text{TP}}{2\cdot \text{TP}+\text{FP}+\text{FN}},$$

6. ROC Curve and AUC

The area under the ROC curve (AUC) measures threshold‐independent discrimination. AUC ranges from 0 to 1, where 0.5 indicates random guessing, 1.0 indicates perfect discrimination, and values below 0.5 indicate worse‐than‐random performance (equivalently, performance above 0.5 after swapping the positive/negative labels).

7. Confusion matrix

The confusion matrix provides an interpretable view of error modes (missed subtle fractures vs. spurious positives) per vertebra, and can be aggregated to the patient level for clinical reporting.

8. Intersection over Union (IoU)

Measures overlap between prediction and ground truth:

(14) $$\mathrm{IoU}\;=\frac{\mathrm{TP}}{\mathrm{TP}+\mathrm{FP}+\mathrm{FN}}\;,$$

### 3.3 Baselines

All baselines share exactly the same preprocessing (raw CT windowed with *WL* = 300 *HU* and *WW* = 1500 *HU*, clipped to [−450,1050] *HU*, linearly rescaled to [0,1], and the same construction of the bone‐likelihood and LoG‐based edge‐enhancement channels), patient‐wise data splits (Section 3.1.2), three‐channel inputs (raw CT, bone‐likelihood, and edge‐enhancement), data augmentations, optimizer and schedule, crop and batch settings, sliding‐window inference with test‐time flips, and the same post‐processing and evaluation code. The segmentation objective for baselines is the combined Dice and cross‐entropy loss unless a method specifies otherwise.

**UNETR**. This model uses a ViT‐style 3D Transformer encoder with patch embeddings and self‐attention, coupled to a CNN decoder via skip connections. We keep a standard “base” configuration and do not add attention gating or geometric regularizers so that differences are attributable to the encoder design.

**Swin UNETR**. This variant replaces vanilla self‐attention with hierarchical 3D Swin blocks that perform windowed and shifted attention with patch merging, followed by a U‐shaped decoder. Training protocol and inputs match UNETR for a controlled comparison.

**nnU‐Net**. The self‐configuring 3D U‐Net is constrained to our fixed spacing and three‐channel inputs. We retain its default deep supervision and the combined Dice and cross‐entropy objective while matching our crop size and batch size to ensure parity.

**3D CNN (plain 3D U‐Net)**. A conventional five‐level encoder‐decoder with residual double‐convolution blocks, group normalization, and transposed‐convolution upsampling. No Transformer modules, attention gates, or geometry‐aware terms are included.

**Proposed Method (segmentation head)**. Our comparison uses only the Stage‐2 network so that metrics reflect segmentation quality. The encoder is initialized from Stage‐1 bone‐window masked‐autoencoder pretraining. The decoder integrates gated attention in the upsampling path to sharpen inter‐vertebral boundaries. The objective augments the combined Dice and cross‐entropy loss with a boundary‐aware term and two geometry‐consistency regularizers that enforce cranio‐caudal ordering and discourage overlaps between adjacent vertebrae. Given the limited number of voxel‐wise labels, we also apply morphology‐consistent self‐training with high‐confidence pseudo‐masks generated on the training split.

Reporting. All methods train for the same number of epochs with early stopping on validation Dice, mixed‐precision training, gradient accumulation when needed, identical inference overlap, and identical post‐processing. We additionally report variants that receive only the raw CT channel, and ablations of our method that remove pretraining, gated attention, or geometry‐aware terms to isolate their contributions.

### 3.4 Quantitative results

#### 3.4.1 Segmentation results

**FIGURE 2:**
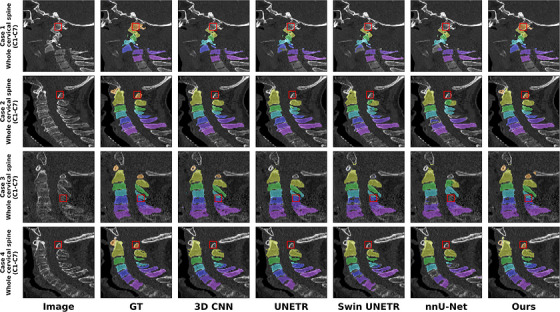
Qualitative comparison of cervical vertebra segmentation on sagittal CT images. Each row corresponds to a representative case with a sagittal slice covering the whole cervical spine (C1–C7), as indicated by the row labels. From left to right, the columns show the input image, GT, and segmentation overlays produced by 3D CNN, UNETR, Swin UNETR, nnU‐Net, and the proposed method. Red boxes indicate representative challenging regions for visual comparison.

Figure 2 presents qualitative vertebral segmentation on sagittal CT across representative cases. From left to right in each row, we show the input CT, the GT labels (C1–C7), and the predictions produced by 3D CNN, UNETR, Swin UNETR, nnU‐Net, and our method. The red boxes highlight challenging regions (e.g., C1–C2 and the cervicothoracic junction), where our method better preserves inter‐vertebral boundaries and reduces leakage into adjacent structures.

Table 2 reports fivefold cross‐validation results under the unified protocol. Besides Dice, we include IoU and HD95 (95th percentile Hausdorff distance, in millimeters). The proposed method attains the best performance on all metrics. Per‐vertebra segmentation performance (Dice/IoU/HD95 for C1–C7) is reported in Table 3. We observe performance variability across vertebral levels, with C1 being more challenging due to its ringlike anatomy and weaker boundary contrast, whereas mid‐to‐lower cervical levels show consistently higher overlap metrics. This level‐wise analysis complements the macro‐averaged results in Table 2 and provides a more granular view of segmentation reliability.

**TABLE 2:** Vertebral segmentation comparison on RSNA (5‐fold CV; mean ± std). All metrics averaged across C1–C7.

Method	Dice (%)	IoU (%)	HD95 (mm)
3D CNN (plain 3D U‐Net)	84.10 ± 1.25	72.6 ± 1.40	2.98 ± 0.25
UNETR	86.02 ± 1.10	75.5 ± 1.25	2.70 ± 0.22
Swin UNETR	86.45 ± 1.05	76.1 ± 1.20	2.62 ± 0.21
nnU‐Net	87.10 ± 0.98	77.2 ± 1.10	2.48 ± 0.20
Proposed method	89.23 ± 0.87	80.6 ± 1.05	2.12 ± 0.18

**TABLE 3:** Per‐vertebra segmentation performance (C1–C7) of SpineMAE on the RSNA cervical spine dataset.

Vertebra level	Dice (%)	IoU (%)	HD95 (mm)
C1	74.58	59.40	2.58
C2	94.08	88.70	1.78
C3	92.57	86.10	1.89
C4	91.92	84.90	2.01
C5	91.00	83.30	2.13
C6	90.64	82.70	2.19
C7	89.82	79.10	2.26
Macro‐average	89.23	80.60	2.12

To attribute the gains, Table 4 ablates the Stage‐2 segmentation head. Removing individual components degrades Dice, IoU, and boundary accuracy (HD95). Training with the pretrained encoder but without our Stage‐2 additions yields performance close to a plain UNETR; removing pretraining pushes results toward a CNN‐only baseline. Model complexity (parameters and FLOPs) for the main comparison methods is summarized in Table 5.

**TABLE 4:** Ablation study on the proposed segmentation head. (5‐fold CV; mean ± std). All metrics averaged across C1–C7.

Variant (relative to full model)	Dice (%)	IoU (%)	HD95 (mm)
Full model (ours)	89.23 ± 0.87	80.6 ± 1.05	2.12 ± 0.18
− Geometry consistency (ordering and overlap)	88.40 ± 0.92	79.3 ± 1.10	2.24 ± 0.19
− Gated attention in upsampling	88.10 ± 1.00	78.8 ± 1.12	2.28 ± 0.20
− Boundary‐aware term	87.85 ± 0.98	78.3 ± 1.14	2.31 ± 0.20
− Structural priors (CT only)	87.20 ± 1.05	77.4 ± 1.20	2.39 ± 0.21
− Self‐training (no pseudo‐labels)	88.00 ± 0.95	78.6 ± 1.13	2.30 ± 0.19
Pretraining only (UNETR‐like)	86.15 ± 1.06	75.7 ± 1.22	2.60 ± 0.22
No pretraining (random init)	84.35 ± 1.22	73.0 ± 1.35	2.88 ± 0.24

**TABLE 5:** Model complexity comparison of segmentation backbones. Parameters are reported in millions (*M*). FLOPs are reported in billions (*G*) under the same input setting used in our segmentation experiments.

Method	Parameters (*M*)	FLOPs (*G*)
3D CNN (plain 3D U‐Net)	16.3	135.2
nnU‐Net	31.2	412.5
Swin UNETR	62.2	104.0
UNETR	92.8	82.6
Proposed method (SpineMAE)	95.4	86.5

#### 3.4.2 Classification results

The vertebra‐level fracture classification performance of the proposed framework was comprehensively evaluated using the segmented vertebral ROIs derived from the previous stage (Figure 2) on the 87 voxel‐labeled studies. Our model achieved strong vertebra‐level fracture classification performance across the cross‐validated test splits, with an AUC of 0.969, sensitivity (recall) of 0.788, and specificity of 0.962, macro‐averaged across vertebral levels (C1–C7). Given the pronounced class imbalance at the vertebra level, we primarily emphasize threshold‐independent discrimination (AUC) and operating characteristics (sensitivity/specificity). For completeness, the corresponding precision and F1‐score at the calibrated operating point were 0.770 and 0.779, respectively.

**FIGURE 3:**
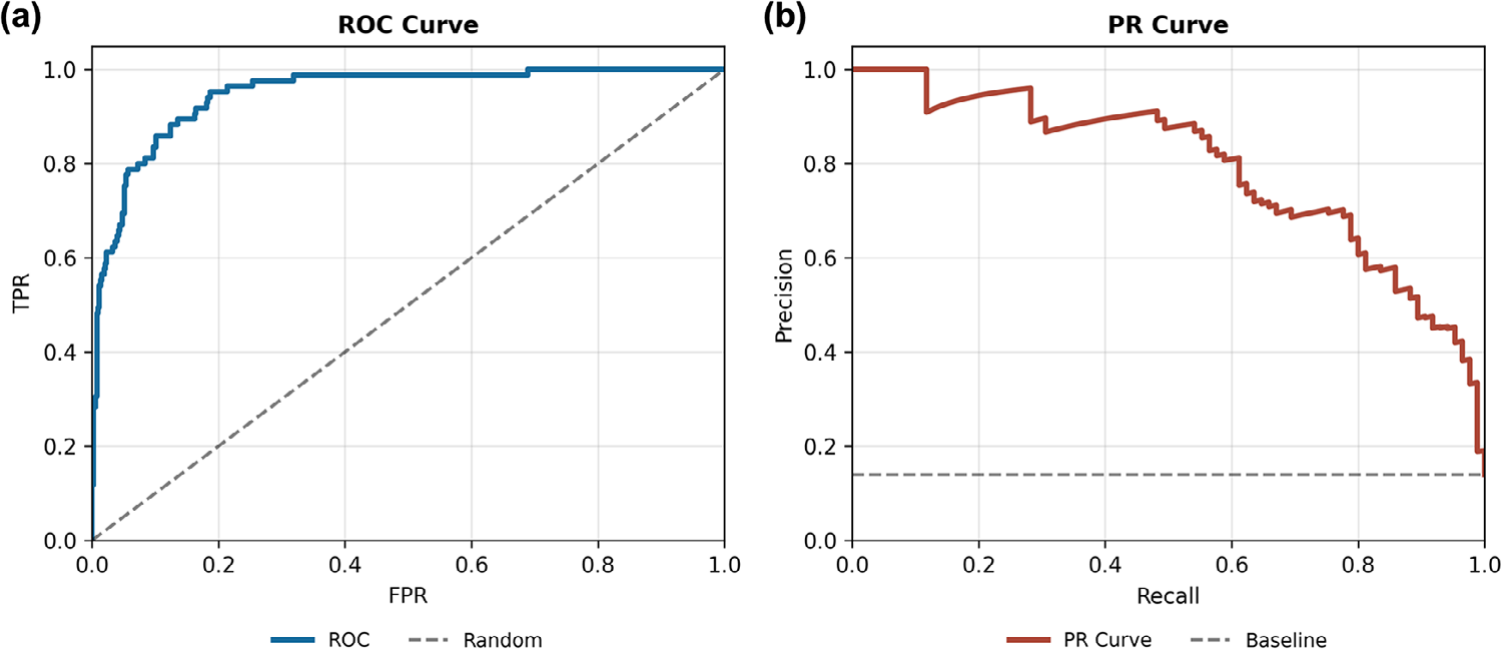
(a) ROC curve and (b) PR curve for vertebra‐level fracture classification.

To assess the classifier's threshold‐independent discriminative performance, Figure 3(a) shows the Receiver Operating Characteristic (ROC) curve, which plots the true positive rate (TPR) against the false positive rate (FPR) across all decision thresholds. The curve maintains a steep ascent near the origin and remains close to the upper‐left boundary, reflecting excellent separation between positive and negative cases. The resulting AUC value (≈0.97) confirms that the model can sustain high sensitivity while controlling false alarms—a desirable property in clinical triage systems.

Figure 3(b) presents the Precision‐Recall (PR) curve, which provides deeper insight into model behavior under class imbalance. The dashed line denotes the positive‐class prevalence baseline (≈14% of vertebrae are fractured). As recall increases, precision gradually decreases (as reflected by the downward trend in the high‐recall range), yet the PR curve remains well above the prevalence baseline across a broad operating region. This indicates that the model improves positive predictive value over a naïve classifier under severe imbalance, while allowing threshold selection to trade sensitivity against false positives depending on clinical requirements.

**FIGURE 4:**
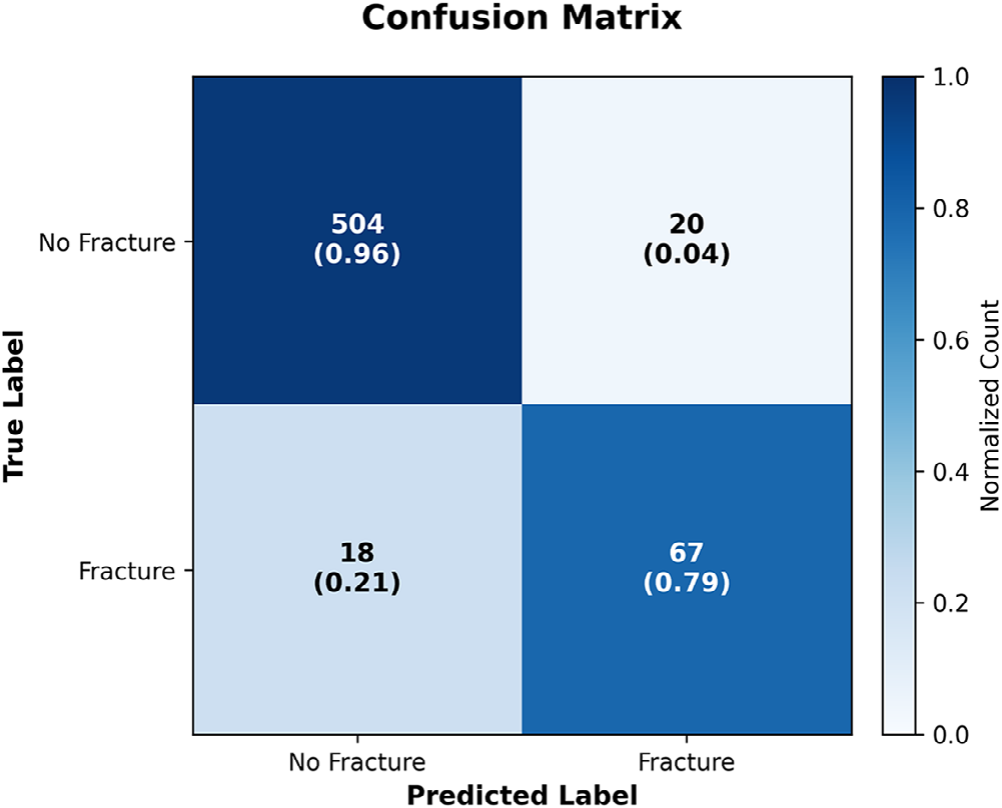
Confusion matrix at the calibrated operating point.

The model's decision outcomes are visualized in Figure 4, which depicts the confusion matrix at the calibrated operating threshold selected to maximize the validation F1‐score. The confusion matrix shows *TN* = 504, *FP* = 20, *FN* = 18, and *TP* = 67. In this imbalanced setting, accuracy can be misleading; therefore, we focus on sensitivity/specificity and AUC when interpreting classifier performance. Importantly, 18 false negatives out of 85 fractured vertebrae correspond to a false‐negative (FN) rate of 21.2% (i.e., sensitivity of 0.788), indicating that missed fractures remain a non‐negligible failure mode at this operating point. Meanwhile, the relatively low false‐positive (FP) count (20) yields a specificity of 0.962. These results highlight the expected sensitivity‐specificity trade‐off, and the decision threshold can be adjusted to prioritize sensitivity when clinically preferred, at the cost of additional false positives.

**FIGURE 5:**
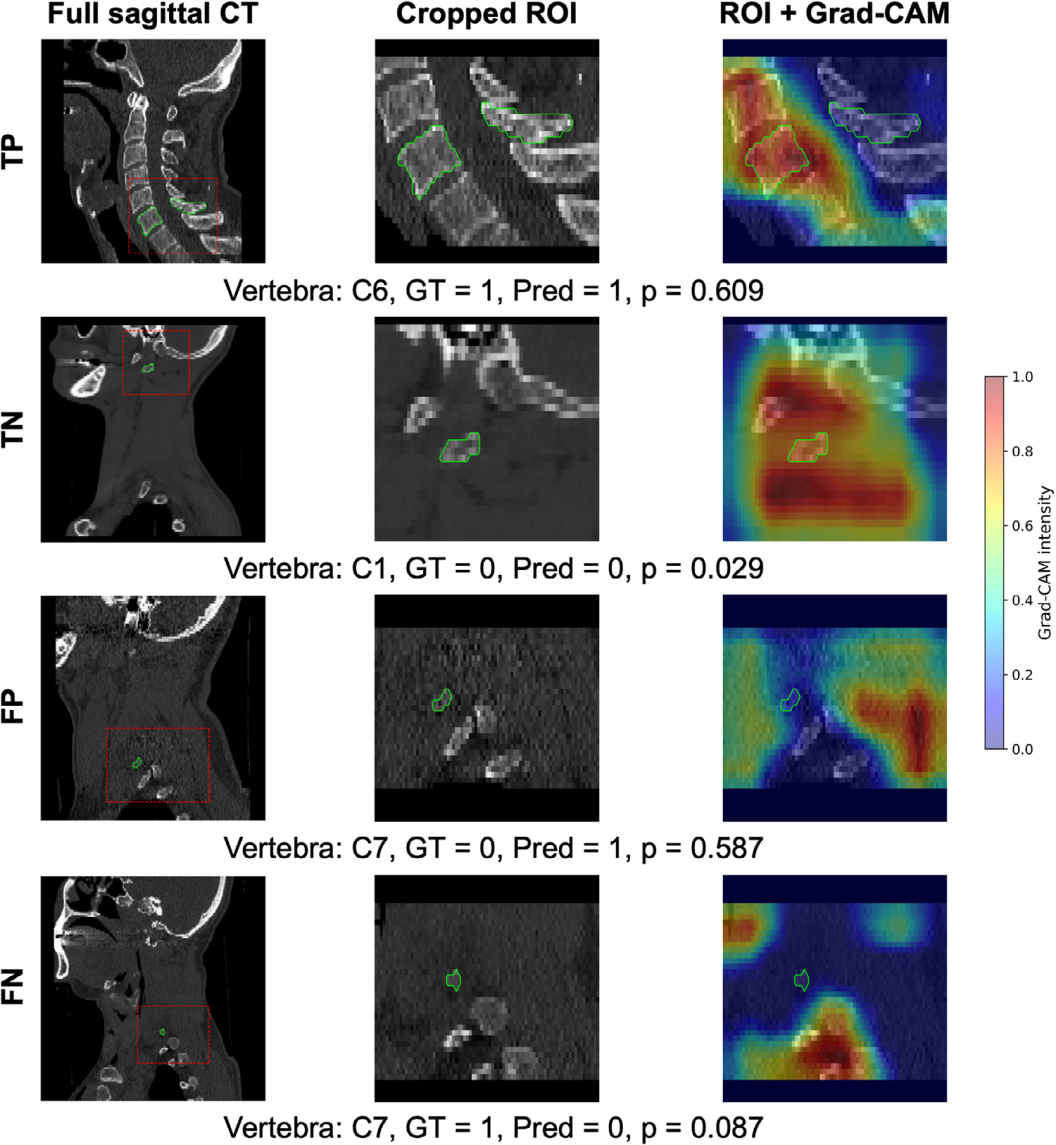
Representative true‐positive (TP), true‐negative (TN), false‐positive (FP), and false‐negative (FN) cases for vertebra‐level fracture classification with Grad‐CAM visualization. Each row shows one representative case. The first column shows the full sagittal CT image, with the source region of the cropped ROI explicitly marked by a red dashed box. The second column shows the cropped ROI, and the third column shows the ROI with Grad‐CAM overlaid. Detailed case information, including vertebral level, ground‐truth label, predicted label, and predicted fracture probability, is provided below each row. All sagittal panels were regenerated using display‐only resampling to 1.0 × 1.0 mm in‐plane resolution to ensure square physical pixels.

To better illustrate the features driving fracture classification, Figure 5 provides representative examples of *TP*, *TN*, *FP*, and *FN* predictions together with Grad‐CAM visualizations. In true‐positive (TP) cases, Grad‐CAM concentrates on cortical regions consistent with fracture‐related appearance changes within the segmented vertebra ROI. In contrast, FP and FN examples show that the model may attend to confounding high‐contrast structures or diffuse regions when fracture cues are subtle or partially visible, highlighting typical failure modes under class imbalance and limited lesion conspicuity.

Finally, Table 6 presents the ablation results analyzing the effects of Stage‐1 pretraining and vertebral level embeddings (position priors). Removing either component leads to consistent degradation in all metrics, confirming that self‐supervised pretraining significantly enhances feature representation, while the inclusion of positional embeddings improves context‐awareness and decision consistency across vertebral levels.

**TABLE 6:** Ablation study on vertebra‐level fracture classification.

Configuration	Accuracy	Precision	Recall	F1	Specificity	AUC
Full model: pretraining + level embedding	0.938	0.770	0.788	0.779	0.962	0.969
Pretraining only (no level embedding)	0.926	0.742	0.774	0.758	0.953	0.961
Level embedding only (no pretraining)	0.914	0.708	0.760	0.733	0.943	0.948
Neither pretraining nor level embedding	0.902	0.681	0.735	0.707	0.934	0.939

To contextualize our results against existing approaches on the RSNA benchmark and prior external validations, we summarize representative published and commercial algorithms in Table 7. Because different studies report performance at different evaluation levels and cohorts, we list the metrics exactly as provided in the original publications.

**TABLE 7:** Comparison with published and commercial cervical spine fracture detection algorithms on the RSNA benchmark and external validation. Patient‐level and vertebra‐level metrics are reported as provided in the original studies; these evaluation levels are not directly comparable.

Method	Evaluation level	Sensitivity	Specificity	AUC
Harper et al.^2^—Competition set	Patient‐level	0.850	0.940	0.950
Harper et al.^2^—External set	Patient‐level	0.860	0.700	0.850
Salehinejad et al. (Deep sequential learning)	Patient‐level	0.772	0.801	–
Voter et al. (Aidoc commercial AI)	Patient‐level	0.550	0.940	–
Small et al. (Aidoc commercial AI)	Patient‐level	0.760	0.970	–
Harper et al.^2^—External set	Vertebra‐level	0.583	0.940	–
SpineMAE (Ours)	Vertebra‐level	0.788	0.962	0.969

Notably, the 0.85 sensitivity reported for the RSNA winning algorithm in Ref.^2^ is a patient‐level metric, where detecting any fractured vertebra is sufficient to count a patient as positive, whereas our primary results are vertebra‐level macro‐averaged across C1–C7, requiring correct localization to the specific vertebra. Ref.^2^ further reports substantially lower and more variable performance when evaluated at the vertebra level on an external set, highlighting that patient‐level sensitivity can mask level‐wise instability. Therefore, Table 7 should be interpreted with attention to evaluation level (patient vs. vertebra), dataset, and operating point.

## 4 DISCUSSION

The proposed framework integrates self‐supervised 3D pretraining, structure‐aware segmentation, and context‐guided classification to enable accurate and anatomically coherent cervical spine fracture analysis. The consistent performance across segmentation and classification suggests that the model captures fine‐grained bone morphology while preserving vertebral‐level spatial relationships, supporting robustness in heterogeneous CT data.

In segmentation, the incorporation of bone‐likelihood and edge priors guides the network toward high‐contrast cortical structures, while the gated attention mechanism refines boundary recovery around complex interfaces. The geometry‐consistency losses, which enforce spatial ordering and reduce overlap between adjacent vertebrae, improve anatomical integrity without additional supervision. Meanwhile, bone‐window self‐supervised pretraining helps the encoder learn shape‐sensitive and boundary‐relevant representations; in particular, the cortex‐biased masking strategy encourages the model to better preserve thin cortical and endplate patterns that are crucial for accurate delineation. From a deployment perspective, baseline backbones differ substantially in parameter count and computational cost, which should be considered alongside accuracy when selecting a model for clinical workflows. Overall, the proposed framework provides a clear accuracy‐efficiency trade‐off and can be chosen according to available computational resources and clinical priorities.

In classification, transferring the pretrained encoder and introducing vertebral‐level embeddings improve discriminative performance and training stability. The ROC analysis indicates strong class separability, while the PR curve—more informative under pronounced class imbalance—remains substantially above the prevalence baseline across a broad range of decision thresholds, despite the expected decrease in precision as recall increases. This behavior reflects the inherent sensitivity‐precision trade‐off and supports operating‐point selection tailored to clinical preferences (e.g., prioritizing sensitivity with an acceptable FP rate). Notably, missed fractures remain a clinically relevant failure mode at the chosen operating point, motivating threshold customization and continued efforts to improve sensitivity for subtle lesions. The ablation results further suggest that pretraining strengthens feature discrimination, whereas vertebral‐level/positional cues inject anatomical context that mitigates cross‐level confusion. Importantly, comparisons to prior work should account for evaluation level: patient‐level sensitivity reported for RSNA challenge systems is not directly comparable to our vertebra‐level macro‐averaged evaluation across C1–C7, which requires correct localization to a specific vertebra. Prior reports^2^ also indicate that level‐wise performance can be unstable when examined per vertebra, highlighting that patient‐level metrics may mask vertebra‐level variability. In this context, our framework achieves strong vertebra‐level operating characteristics while additionally providing anatomically coherent vertebra masks for localization and visual verification, which improves interpretability beyond black box fracture classifiers.

To improve interpretability, we examined representative true/false predictions using gradient‐based saliency on vertebra ROIs. Correct predictions typically emphasize cortical and endplate regions that are clinically relevant for fracture assessment, whereas false positives and false negatives often arise when cues are subtle, partially visible, or confounded by other high‐contrast anatomical structures and artifacts. This observation motivates future work on hard‐negative mining and robustness to subtle lesions.

Clinically, this framework offers a modular and interpretable solution for automated cervical spine assessment, supporting both visual validation via segmentation maps and quantitative fracture likelihoods for each vertebra. However, the study still faces limitations: the availability of voxel‐labeled cases is relatively limited, which may constrain generalization, and the current binary fracture formulation does not capture fracture subtype or severity. Future work will extend this pipeline toward semi‐supervised learning, domain adaptation, and fracture categorization to improve clinical applicability.

In summary, combining self‐supervised 3D representation learning with anatomy‐aware modeling provides a promising pathway toward reliable and explainable spine fracture AI systems, capable of assisting radiologists with both detection and localization in routine clinical workflows.

## 5 CONCLUSION

In this study, we proposed a three‐stage framework combining self‐supervised 3D pretraining, structure‐aware segmentation, and context‐guided fracture classification for automated cervical spine analysis. The model effectively captures bone morphology and anatomical relationships, achieving accurate vertebral segmentation and reliable vertebra‐level fracture prediction. Experiments on the RSNA dataset show consistent improvements over both CNN‐ and transformer‐based baselines, with a Dice score of 89.23% and fracture classification performance of AUC = 0.969, sensitivity = 0.788, and specificity = 0.962 (macro‐averaged across C1–C7). The results further suggest that bone‐window pretraining enhances representation quality, while vertebral‐level embeddings improve contextual understanding and decision consistency across vertebrae. Overall, the proposed method provides a robust and interpretable solution for cervical spine fracture analysis, offering potential for integration into clinical diagnostic workflows.

## AUTHOR CONTRIBUTIONS

All authors participated in study design, data collection, analysis, and manuscript preparation.

## CONFLICT OF INTEREST STATEMENT

The authors declare no conflicts of interest.

## ETHICS STATEMENT

This study used a publicly available, de‐identified dataset and involved no direct interaction with human participants or access to identifiable private information. Therefore, institutional review board approval and informed consent were not required.

## Data Availability

The data used in this study are publicly available from the RSNA Cervical Spine Fracture Detection dataset. All derived models, code, and pretrained weights will be made available upon reasonable request to the corresponding authors. No additional private dataset was used in this study.
